# Alternative diagnoses and demographics associated with a raised quantitative faecal immunochemical test in symptomatic patients

**DOI:** 10.1177/00045632221076771

**Published:** 2022-03-03

**Authors:** Mark S Johnstone, Gillian Miller, Grace Pang, Paul Burton, Georgios Kourounis, Jack Winter, Emilia Crighton, David Mansouri, Paul Witherspoon, Karen Smith, Stephen T McSorley

**Affiliations:** 1 Academic Unit of Surgery, School of Medicine, 3526 University of Glasgow, Glasgow, UK; 2 362869 eHealth, Corporate Services, Business Intelligence, NHS Greater Glasgow and Clyde, Glasgow, UK; 3 Department of Gastroenterology, 362869 Glasgow Royal Infirmary, NHS Greater Glasgow and Clyde, Glasgow, UK; 4 Public Health, Health Service, Public Health Screening, NHS Greater Glasgow and Clyde, Glasgow, UK; 5 Department of Coloproctology, 362869 Glasgow Royal Infirmary, NHS Greater Glasgow and Clyde, Glasgow, UK; 6 Department of Colorectal Surgery, 427872 Queen Elizabeth University Hospital, NHS Greater Glasgow and Clyde, Glasgow, UK; 7 Department of Clinical Biochemistry, 362869 Glasgow Royal Infirmary, NHS Greater Glasgow and Clyde, Glasgow, UK

**Keywords:** Faecal, immunochemical, test, haemoglobin, colorectal, cancer, symptomatic

## Abstract

**Background:**

The faecal immunochemical test (FIT) has proven utility for colorectal cancer detection in symptomatic patients. However, most patients with a raised faecal haemoglobin (f-Hb) do not have colorectal cancer. We investigated alternative diagnoses and demographics associated with a raised f-Hb in symptomatic patients.

**Methods:**

A retrospective, observational study was performed of patients with FIT submitted between August 2018 to January 2019 in NHS Greater Glasgow and Clyde followed by colonoscopy. Colonoscopy/pathology reports were searched for alternative diagnoses. Covariables were compared using the χ^2^ test. Multivariate binary logistic regression identified independent predictors of a raised f-Hb.

**Results:**

1272 patients were included. In addition to colorectal cancer (odds ratio (OR), 9.27 (95% confidence interval (CI): 3.61–23.83; *p* < 0.001)), older age (OR, 1.52 (95% CI: 1.00–2.32; *p* = 0.05)), deprivation (OR, 1.54 (95% CI: 1.21–1.94; *p* < 0.001)), oral anticoagulants (OR, 1.78 (95% CI: 1.01–3.15; *p* = 0.046)), rectal bleeding (OR, 1.47 (95% CI: 1.15–1.88; *p* = 0.002)), advanced adenoma (OR, 7.52 (95% CI: 3.90–14.49; *p* < 0.001)), non-advanced polyps (OR, 1.78 (95% CI: 1.33–2.38; *p* < 0.001)) and inflammatory bowel disease (IBD) (OR, 4.19 (95% CI: 2.17–8.07; *p* < 0.001)) independently predicted raised f-Hb. Deprivation (Scottish Index of Multiple Deprivation (SIMD) 1-2: OR, 2.13 (95% CI: 1.38–3.29; *p* = 0.001)) independently predicted a raised f-Hb in patients with no pathology found at colonoscopy.

**Conclusions:**

An elevated f-Hb is independently associated with older age, deprivation, anticoagulants, rectal bleeding, advanced adenoma, non-advanced polyps and IBD in symptomatic patients. Deprivation is associated with a raised f-Hb in the absence of pathology. This must be considered when utilising FIT in symptomatic patients.

## Introduction

The faecal immunochemical test (FIT) has proven utility for colorectal cancer detection in symptomatic patients, sensitivity and specificity reportedly ranging from 85% to 100% and 56%–91%, respectively, at a threshold of ≥ 10 μg Hb/g faeces.^1–8^ The National Institute for Health and Care Excellence (NICE) now recommends FIT be used in patients with high-risk symptoms that may trigger an urgent suspected cancer referral (NG12)^9^ and in those with lower risk symptoms (DG30).^10^ In response, a number of UK health boards/trusts have introduced universal FIT submission as part of symptomatic lower GI referral pathways.^11^ However, most patients with a raised f-Hb will not have a colorectal cancer. A raised f-Hb in symptomatic patients has been correlated with advanced adenomas and inflammatory bowel disease.^12–15^ Indeed, there is evidence that FIT can be used as a marker of disease activity in ulcerative colitis^16–20^ and colonic Crohn’s^21^ as an adjunct to faecal calprotectin. Additionally, higher FIT positivity in the context of bowel cancer screening has been independently associated with older age, male sex, deprivation, aspirin, non-steroidal anti-inflammatory drugs (NSAIDs), oral anticoagulants, proton pump inhibitors (PPIs), antibiotics and smoking,^22–25^ and false positivity has been related to younger age, female sex, smoking, high BMI, successive screening, aspirin, NSAIDs, PPIs, antibiotics, laxatives, non-advanced adenomas, diverticular disease, haemorrhoids, anal fissures and peptic ulceration.^25–32^

To date, no studies have examined demographics which independently predict a raised f-Hb in symptomatic patients and very few have explored non-cancer diagnoses which correlate with f-Hb. We aimed to establish demographics and alternative pathologies associated with a raised f-Hb in a cohort of symptomatic patients.

## Methods

A retrospective, observational study was conducted to include all adult (≥ 16 years) patients with an FIT submitted from primary care between August 2018 and January 2019 in NHS Greater Glasgow and Clyde (period in which FIT was introduced to local referral pathways).

### Faecal immunochemical test specimen collection and handling

FIT collection kits containing a single FIT collection device (EXTEL HEMO AUTO MC Collection Picker, Minaris Medical Co, Ltd, Tokyo, Japan, supplied by Alpha Labs Ltd, Eastleigh, Hants, UK) with accompanying pictorial instructions, and return envelopes were supplied to general practitioners (GPs) as an adjunct to guide symptomatic lower gastrointestinal (GI) referrals. The collection device is in the form of a picker with an internal septum which removes excess faeces and provides a consistent 2-mg sample, which is inserted into a vial containing 2 mL of buffer following collection. Patients were asked to collect a single faecal sample and return to their GP practice as soon as possible. The kits were transported at ambient temperature via routine specimen collection services and stored at 4°C prior to analysis in a single centralised laboratory (Stobhill Hospital, Glasgow).

### Faecal immunochemical test analysis

Analysis was carried out on a HM-KACKarc system (Minaris Medical Co, Ltd), once for each sample, Monday to Friday so most samples were analysed on day of receipt. The manufacturers quote a limit of detection of 2 μg/g, a limit of quantification of 7 μg/g and an upper measurement limit of 400 μg/g. Specimens with f-Hb concentrations above this limit were not diluted and re-analysed.

### Faecal immunochemical test result quality management

All biomedical science staff are Health Care and Professionals Council (HCPC) registered and undergo training and local competency assessment prior to operating the HM-KACKarc analyser. The analyser is calibrated daily. There are two internal quality controls (IQCs): EXTEL HEMO AUTO HS Low IQC and EXTEL HEMO AUTO HS High IQC. West guard rule criteria are used for acceptance or rejection of analytical runs. IQC performance is reviewed monthly and manufacturers’ targets are refined when appropriate. Current performance: low QC mean = 23.2 μg/g, CV = 8.3%, high QC mean = 90.7 μg/g and CV 6.6%. The laboratory participates in external quality assessment via the United Kingdom National External Quality Assessment Service (UK NEQAS) on a monthly basis, with good recent performance scores.

### Faecal immunochemical test result handling

Faecal immunochemical test results are electronically transferred from the analyser into the Laboratory Information Management System (LIMS) and patient record. Any result with an error code is investigated and the appropriate result entered manually. Finally, results are electronically reported to the requesting GP with a range of ≤ 9 μg/g to ≥ 400 μg/g. Samples ≥ 10 μg/g were defined as raised as per the NICE DG30 guidance.^10^

### Patient identification and data collection

To identify study participants, a search of the clinical biochemistry repository was conducted. To obtain patient demographics and outcomes, cross-referencing of the SCI Store, SCI Gateway, Unisoft, Clinical Portal and Managed Clinical Networks (MCN) Cancer Registry was performed with the community health index (CHI) number used as the linkage variable. Caldicott guardian approval was given by NHS GG&C to safeguard the record linkage with ethical approval waived for the purposes of service development. A search of SCI store (Scottish Care Information Store Version 8.5) allowed identification of patient demographics and blood results. SCI Gateway (Scottish Care Information Gateway R 20.0) was searched to identify referral letters from primary care to general surgery or gastroenterology within 6 weeks of FIT collection. These letters were manually screened to identify lower GI symptoms and coded as rectal bleeding, persistent diarrhoea, other change in bowel habit, weight loss, abdominal pain, anal pain, faecal soiling, rectal mass and abdominal mass. Referral letters also identified patient co-morbidity. Unisoft (Unisoft Medical Systems GI Reporting Tool) identified all patients who underwent a colonoscopy following FIT collection. Each colonoscopy record and any accompanying pathology records were screened manually to identify lower GI diagnoses and coded as cancer, advanced adenoma(s), any advanced polyp(s), non-advanced polyp(s), inflammatory bowel disease, other inflammation (infective colitis, collagenous colitis, lymphocytic colitis and inflammatory polyps), diverticulosis, haemorrhoids, angiodysplasia/telangiectasia, radiation proctitis, other malignancy (anal squamous cell carcinoma and rectal lymphoma), melanosis coli, anal fissure or fistula, rectal prolapse, fibroepithelial anal polyp and lipoma. Advanced adenomas were defined as those ≥ 10 mm or with the presence of high-grade dysplasia. Advanced polyps were defined as advanced adenomas or advanced serrated polyps ≥ 10 mm or with the presence of any grade of dysplasia as per The British Society of Gastroenterology/Association of Coloproctology of Great Britain and Ireland surveillance guidelines.^33^ The MCN cancer registry was searched to identify all new diagnoses of colorectal cancer up to November 2020. For the purposes of analysis, patients were divided into those with significant bowel disease (cancer, advanced adenoma, advanced polyp, ≥5 non-advanced polyps or inflammatory bowel disease), other bowel disease (any other positive finding at colonoscopy) and no pathology (entirely normal colonoscopy).

### Statistical analysis

FIT results were grouped by f-Hb concentrations of < 10 μg/g, 10–149 μg/g, 150–399 μg/g and >399 μg/g.^34^ Patients were defined as anaemic (male haemoglobin (Hb) < 130 mg/L and female Hb < 120 mg/L) and iron deficient (ferritin <15 μg/L) based on World Health Organisation (WHO) guidelines.^35^ Covariables were compared using crosstabulation and the *χ*^2^ test or Fisher’s exact test. A value of *p* < 0.05 was considered statistically significant. To identify covariables which independently predicted a raised f-Hb, univariate followed by multivariate binary logistic regression was performed. Covariables of interest from the *χ*^2^ analysis were carried into the regression analysis. Variables found to be significant on *χ*^2^ analysis but where there were insufficient numbers for regression analysis were excluded. For the purposes of regression analysis, FIT was converted to a binary variable: normal (f-Hb < 10 μg/g) vs. raised (f-Hb ≥10 μg/g). This allowed calculation of odds ratios (ORs) and 95% confidence intervals (95% CI). Covariables significant on univariate analysis (*p*< 0.05) were entered into a multivariate model using the backwards conditional method in which variables with a significance of *p* < 0.1 were removed in a stepwise fashion. The same process was then performed in turn only for those patients with significant bowel disease, other bowel disease and no pathology. Statistical analysis was performed using SPSS software (SPSS Inc., Chicago, Illinois, USA).

## Results

4968 patients had a FIT sample submitted from primary care between August 2018 and January 2019 in NHS GG&C. Of these, 2434 patients were subsequently referred to general surgery or gastroenterology and 1327 of those underwent colonoscopy. Of those who underwent colonoscopy, 572 (43.1%) had f-Hb < 10 μg/g and 700 (52.8%) f-Hb ≥ 10 μg/g, with 430 (32.4%) between 10 and 149 μg/g, 89 (6.7%) between 150 and 399 μg/g and 181 (13.6%) ≥ 400 μg/g. 55 (4.1%) samples could not be processed by the laboratory due to faecal contamination, expired collection device or insufficient patient identification, and were not repeated. These patients were excluded from the final analysis, leaving a total of 1272 patients who underwent colonoscopy and had a valid FIT.

Median age of these 1272 patients was 60 years (range, 17–94), with 558 (43.9%) male and 714 (56.1%) females. 561 (44.1%) patients reported rectal bleeding; 348 (27.4%), persistent diarrhoea; 602 (47.3%), other change in bowel habit; 214 (16.8%), weight loss; 383 (30.1%) abdominal pain; 33 (2.6%), anal pain; 77 (6.1%), faecal soiling; 25 (2.0%), rectal mass; and 31 (2.4%), abdominal mass.

### Comparison of demographics by f-Hb concentration

Table 1 shows a comparison of demographics by f-Hb concentration. Having a raised f-Hb was associated with either being below (< 50 years) or above (≥ 75 years) the Scottish Bowel Screening Programme age (50–74 years) (*p* < 0.001). There was no association between f-Hb and sex (*p* = 0.08). Deprivation was associated with a higher f-Hb (*p* = 0.004). No individual co-morbidity was associated with a raised f-Hb; however, the presence of diabetes (*p* = 0.015) or hypertension (*p* = 0.004) seemed to be mildly protective. Patients on oral anticoagulants or PPIs were significantly more likely to have a raised f-Hb (*p* = 0.017) or raised f-Hb between 10 and 399 μg/g (*p* = 0.007), respectively. Patients self-reporting rectal bleeding were more likely to have a raised f-Hb (*p* < 0.001), while a history of persistent diarrhoea (*p* < 0.001), other change in bowel habit (*p* = 0.004) or faecal soiling (*p* < 0.001) were associated with a lower f-Hb.

**Table 1.** Comparison of demographics by f-Hb concentration.

			f-Hb (μg Hb/g faeces)				*p*
		All	< 10	10–149	150–399	≥ 400	
N		1272	572	430	89	181	
Age (years)	Median (range)	60 (17–94)	58 (17–88)	63 (19–92)	56 (19–94)	56 (17–90)	< 0.001
	< 50	318 (25.0%)	142 (24.8%)	82 (19.1%)	32 (36.0%)	62 (34.3%)	
	50–74	759 (59.7%)	370 (64.7%)	259 (60.2%)	44 (49.4%)	86 (47.5%)	
	≥ 75	195 (15.3%)	60 (10.5%)	89 (20.7%)	13 (14.6%)	33 (18.2%)	
Sex	Male	558 (43.9%)	235 (41.1%)	188 (43.7%)	48 (53.9%)	87 (48.1%)	0.080
	Female	714 (56.1%)	337 (58.9%)	242 (56.3%)	41 (46.1%)	94 (51.9%)	
Scottish Index of Multiple Deprivation	Non-deprived (SIMD 3–5)	627 (49.3%)	314 (54.9%)	193 (44.9%)	37 (41.6%)	83 (45.9%)	0.004
	Deprived (SIMD 1–2)	645 (50.7%)	258 (45.1%)	237 (55.1%)	52 (58.4%)	98 (54.1%)	
Co-morbidity	Asthma	163 (12.8%)	71 (12.4%)	59 (13.7%)	14 (15.7%)	19 (10.5%)	0.584
	COPD	76 (6.0%)	31 (5.4%)	38 (8.8%)	5 (5.6%)	2 (1.1%)	0.003
	Diabetes	157 (12.3%)	82 (14.3%)	57 (13.3%)	6 (6.7%)	12 (6.6%)	0.015
	Hypertension	109 (8.6%)	65 (11.4%)	33 (7.7%)	4 (4.5%)	7 (3.9%)	0.004
	IHD	150 (11.8%)	60 (10.5%)	61 (14.2%)	9 (10.1%)	20 (11.0%)	0.303
	Cerebrovascular disease	42 (3.3%)	27 (4.7%)	9 (2.1%)	2 (2.2%)	4 (2.2%)	0.087
	PVD	9 (0.7%)	4 (0.7%)	2 (0.5%)	2 (2.2%)	1 (0.6%)	0.331
	IBD (prior diagnosis)	6 (0.5%)	3 (0.5%)	2 (0.5%)	0 (0.0%)	1 (0.6%)	0.923
	CRC (prior diagnosis)	8 (0.6%)	3 (0.5%)	5 (1.2%)	0 (0.0%)	0 (0.0%)	0.287
Medication	Aspirin	268 (21.1%)	116 (20.3%)	100 (23.3%)	18 (20.2%)	34 (18.8%)	0.561
	NSAIDs	166 (13.1%)	77 (13.5%)	49 (11.4%)	14 (15.7%)	26 (14.4%)	0.580
	Clopidogrel/Ticagrelor	58 (4.6%)	26 (4.5%)	18 (4.2%)	5 (5.6%)	9 (5.0%)	0.932
	Oral anticoagulants	69 (5.4%)	20 (3.5%)	26 (6.0%)	9 (10.1%)	14 (7.7%)	0.017
	ACE inhibitors	259 (20.4%)	124 (21.7%)	86 (20.0%)	14 (15.7%)	35 (19.3%)	0.585
	Statins	364 (28.6%)	150 (26.2%)	134 (31.2%)	28 (31.5%)	52 (28.7%)	0.345
	PPI	606 (47.6%)	257 (44.9%)	228 (53.0%)	48 (53.9%)	73 (40.3%)	0.007
	H2 antagonists	39 (3.1%)	16 (2.8%)	14 (3.3%)	5 (5.6%)	4 (2.2%)	0.460
	Metformin	76 (6.0%)	39 (6.8%)	26 (6.1%)	6 (6.7%)	5 (2.8%)	0.249
	Anti-spasmodics	315 (24.8%)	156 (27.3%)	98 (22.8%)	21 (23.6%)	40 (22.1%)	0.310
Symptoms	Rectal bleeding	561 (44.1%)	221 (38.6%)	160 (37.2%)	51 (57.3%)	129 (71.3%)	< 0.001
	Persistent diarrhoea	348 (27.4%)	195 (34.1%)	88 (20.5%)	21 (23.6%)	44 (24.3%)	< 0.001
	Other change in bowel habit	602 (47.3%)	284 (49.7%)	213 (49.5%)	42 (47.2%)	63 (34.8%)	0.004
	Weight loss	214 (16.8%)	108 (18.9%)	73 (17.0%)	11 (12.4%)	22 (12.2%)	0.120
	Abdominal pain	383 (30.1%)	189 (33.0%)	123 (28.6%)	27 (30.3%)	44 (24.3%)	0.127
	Anal pain	33 (2.6%)	14 (2.4%)	9 (2.1%)	2 (2.2%)	8 (4.4%)	0.406
	Faecal soiling	77 (6.1%)	51 (8.9%)	17 (4.0%)	6 (6.7%)	3 (1.7%)	< 0.001
	Rectal mass	25 (2.0%)	17 (3.0%)	7 (1.6%)	0 (0.0%)	1 (0.6%)	0.074
	Abdominal mass	31 (2.4%)	12 (2.1%)	12 (2.8%)	3 (3.4%)	4 (2.2%)	0.833
Anaemia	Anaemia^a^	246 (21.8%)	106 (20.9%)	74 (19.6%)	22 (26.8%)	44 (26.8%)	0.173
	Iron deficiency anaemia^b^	91 (8.1%)	46 (9.2%)	21 (5.6%)	4 (4.9%)	20 (12.3%)	0.032

^a^Missing data for 141 (11.1%) patients.

^b^Data missing for 153 (12.0%) patients.

### Cancer cases

With a median 23-month (range, 21–25) follow-up, 54 patients were diagnosed with a colorectal cancer. 5 (9.3%) had a f-Hb < 10 μg/g; 9 (16.7%), between 10 and 149 μg/g; 7 (13.0%), between 150 and 399 μg/g; and 33 (61.1%), ≥ 400 μg/g.

### Alternative colonoscopic diagnoses associated with a raised f-Hb

Table 2 shows a comparison of colonoscopic/pathology findings by f-Hb concentration. As well as being strongly associated with cancer (*p* < 0.001), a raised f-Hb also correlated with the risk of advanced adenoma (*p* < 0.001), any advanced polyp (*p* < 0.001), non-advanced polyps (*p* < 0.001), inflammatory bowel disease (*p* < 0.001) and other malignancy (anal SCC or rectal lymphoma, *p* < 0.001). There was also a correlation with diverticulosis (*p* < 0.001) although this was predominantly associated with a mildly raised f-Hb (10–149 μg/g). Raised f-Hb was associated with having any pathology found at colonoscopy (*p* < 0.001); although of interest, 142 (20.3%) patients with a raised f-Hb had a completely normal colonoscopy, including 28 (15.5%) with a f-Hb ≥ 400 μg/g.

**Table 2.** Comparison of colonoscopic/pathology findings by f-Hb concentration.

		f-Hb (μg Hb/g faeces)				*p*
	All	< 10	10–149	150–399	≥ 400	
N	1272	572	430	89	181	
*Significant bowel disease*	223 (17.5%)	36 (6.3%)	71 (16.5%)	34 (38.2%)	82 (45.3%)	< 0.001
Colorectal cancer	54 (4.2%)	5 (0.9%)	9 (2.1%)	7 (7.9%)	33 (18.2%)	< 0.001
Advanced adenoma	93 (7.3%)	11 (1.9%)	42 (9.8%)	14 (15.7%)	26 (14.4%)	< 0.001
Any advanced polyp	99 (7.8%)	15 (2.6%)	43 (10.0%)	15 (16.9%)	26 (14.4%)	< 0.001
≥ 5 polyps	33 (2.6%)	6 (1.0%)	18 (4.2%)	5 (5.6%)	4 (2.2%)	0.005
IBD	66 (5.2%)	12 (2.1%)	14 (3.3%)	11 (12.4%)	29 (16.0%)	< 0.001
*Other pathology*	682 (53.6%)	311 (54.4%)	261 (60.7%)	39 (43.8%)	71 (39.2%)	< 0.001
Non-advanced polyp	271 (21.3%)	104 (18.2%)	122 (28.4%)	16 (18.0%)	29 (16.0%)	< 0.001
Other inflammation	41 (3.2%)	20 (3.5%)	11 (2.6%)	4 (4.5%)	6 (3.3%)	0.750
Diverticulosis	408 (32.1%)	172 (30.1%)	169 (39.3%)	22 (24.7%)	45 (24.9%)	< 0.001
Haemorrhoids	197 (15.5%)	100 (17.5%)	56 (13.0%)	14 (15.7%)	27 (14.9%)	0.286
Angiodysplasia/Telangiectasia	10 (0.8%)	3 (0.5%)	5 (1.2%)	0 (0.0%)	2 (1.1%)	0.527
Radiation proctitis	12 (0.9%)	1 (0.2%)	8 (1.9%)	1 (1.1%)	2 (1.1%)	0.056
Anal SCC or rectal lymphoma	4 (0.3%)	0 (0.0%)	0 (0.0%)	0 (0.0%)	4 (2.2%)	< 0.001
Melanosis coli	11 (0.9%)	4 (0.7%)	5 (1.2%)	1 (1.1%)	1 (0.6%)	0.825
Anal fissure/Fistula	8 (0.6%)	4 (0.7%)	1 (0.2%)	0 (0.0%)	3 (1.7%)	0.191
Rectal prolapse	3 (0.2%)	0 (0.0%)	2 (0.5%)	1 (1.1%)	0 (0.0%)	0.126
Fibroepithelial anal polyp	14 (1.1%)	3 (0.5%)	7 (1.6%)	3 (3.4%)	1 (0.6%)	0.056
Lipoma	4 (0.3%)	3 (0.5%)	1 (0.2%)	0 (0.0%)	0 (0.0%)	0.626
*No pathology*	367 (28.9%)	225 (39.3%)	98 (22.8%)	16 (18.0%)	28 (15.5%)	< 0.001

### Raised f-Hb Binary logistic regression

12 variables were chosen for binary logistic regression: age, sex, SIMD, oral anticoagulants, PPI, rectal bleeding, colorectal cancer, advanced adenoma, any advanced polyp, any non-advanced polyp, inflammatory bowel disease and diverticulosis (Table 3). While anal squamous cell carcinoma or rectal lymphoma was found to be significant in *χ*^2^ analysis, the absolute number of cases was very small (*n* = 4) and this could not be included in regression analysis. On univariate analysis older age (≥75 years: OR, 1.82 (95% CI: 1.25–2.64; *p* = 0.002)), deprivation (SIMD 12: OR, 1.51 (95% CI: 1.21–1.88; *p* < 0.001)), oral anticoagulants (OR, 1.82 (95% CI: 1.02–3.27; *p* = 0.045)), rectal bleeding (OR, 1.50 (95% CI: 1.20–1.88; *p* < 0.001)), colorectal cancer (OR, 8.54 (95% CI: 3.38–21.57; *p* < 0.001)), advanced adenoma (OR, 6.68 (95% CI: 3.57–12.83; *p* < 0.001)), any advanced polyp (OR, 5.06 (95% CI: 2.89–8.88; *p* < 0.001)), non-advanced polyps (OR, 1.41 (95% CI: 1.07–1.86; *p* = 0.014)) and inflammatory bowel disease (OR, 3.90 (95% CI: 2.07–7.37; *p* < 0.001)) correlated with a raised f-Hb. On multivariate analysis, older age (≥ 75 years: OR, 1.52 (95% CI: 1.00–2.32; *p* = 0.050)), deprivation (SIMD 12: OR, 1.54 (95% CI: 1.21–1.94; *p* < 0.001)), oral anticoagulants (OR, 1.78 (95% CI: 1.01–3.15; *p* = 0.046)), rectal bleeding (OR, 1.47 (95% CI: 1.15–1.88; *p* = 0.002)), colorectal cancer (OR, 9.27 (95% CI: 3.61–23.83; *p* < 0.001)), advanced adenoma (OR, 7.52 (95% CI: 3.90–14.49; *p* < 0.001)), non-advanced polyps (OR, 1.78 (95% CI: 1.33–2.38; *p* < 0.001)) and inflammatory bowel disease (OR, 4.19 (95% CI: 2.17–8.07; *p* < 0.001)) retained significance as independent predictors of a raised f-Hb.

**Table 3.** Univariate and multivariate binary logistic regression of factors associated with f-Hb ≥ 10 μg/g.

		Univariate			Multivariate		
		OR	95% CI	*p*	OR	95% CI	*p*
Age (years)	< 50	1.00			1.00		
	50–74	0.85	0.65–1.10	0.220	0.82	0.61–1.09	0.163
	≥ 75	1.82	1.25–2.64	0.002	1.52	1.00–2.32	0.050
Sex	Male	1.00					
	Female	0.81	0.65–1.02	0.071			
Scottish Index of Multiple Deprivation	Non-deprived (SIMD 3–5)	1.00			1.00		
	Deprived (SIMD 1–2)	1.51	1.21–1.88	< 0.001	1.54	1.21–1.94	< 0.001
Oral anticoagulants	No	1.00			1.00		
	Yes	1.82	1.02–3.27	0.045	1.78	1.01–3.15	0.046
PPI	No	1.00					
	Yes	1.22	0.98–1.52	0.080			
Rectal bleeding	No	1.00			1.00		
	Yes	1.50	1.20–1.88	< 0.001	1.47	1.15–1.88	0.002
Colorectal cancer	No	1.00			1.00		
	Yes	8.54	3.38–21.57	< 0.001	9.27	3.61–23.83	< 0.001
Advanced adenoma	No	1.00			1.00		
	Yes	6.77	3.57–12.83	< 0.001	7.52	3.90–14.49	< 0.001
Any advanced polyp	No	1.00			–		
	Yes	5.06	2.89–8.88	< 0.001	–	–	0.484
Any non-advanced polyp	No	1.00			1.00		
	Yes	1.41	1.07–1.86	0.014	1.78	1.33–2.38	< 0.001
Inflammatory bowel disease	No	1.00			1.00		
	Yes	3.90	2.07–7.37	<0.001	4.19	2.17–8.07	< 0.001
Diverticulosis	No	1.00					
	Yes	1.18	0.93–1.50	0.166			

### SBD, other pathology and no pathology

Next, patients were divided into those with significant bowel disease, other pathology and no pathology. A comparison of these three groups by f-Hb concentration can be seen in Table 2. 223 patients were found to have cancer, advanced adenoma, advanced polyps, ≥5 polyps or inflammatory bowel disease (significant bowel disease). 36 (16.1%) had f-Hb < 10 μg/g; 71 (31.8%), 10–149 μg/g; 34 (15.2%), 150–399 μg/g; and 82 (36.8%), ≥ 400 μg/g. 682 patients were found to have other bowel disease. 311 (45.6%) had f-Hb < 10 μg/g; 261 (38.3%), 10–149 μg/g; 39 (5.7%), 150–399 μg/g; and 71 (10.4%), ≥ 400 μg/g. 367 had no pathology found at colonoscopy. 225 (61.3%) had f-Hb < 10 μg/g; 98 (26.7%), 10–149 μg/g; 16 (4.4%), 150–399 μg/g; and 28 (7.6%), ≥ 400 μg/g. There was a highly significant association between f-Hb concentration and increasing ‘severity’ of colonoscopic findings from no pathology to other pathology to significant bowel disease (*p* < 0.001).

### Demographics Associated with Raised f-Hb in those with SBD, Other Pathology and No Pathology Binary Logistic Regression

6 demographics were chosen for binary logistic regression: age, sex, SIMD, oral anticoagulants, PPI and rectal bleeding (Table 4). For those patients with significant bowel disease, only rectal bleeding (OR, 3.63 (95% CI: 1.66–7.97; *p* = 0.001) correlated with a raised f-Hb on univariate analysis. Multivariate analysis was therefore not performed. For those patients with other bowel disease, only PPI use (OR, 1.60 (95% CI: 1.15–2.11; *p* = 0.004)) correlated with a raised f-Hb on univariate analysis. Again, multivariate analysis could not be performed. For those with no pathology, bowel screening age (50–74 years) (OR, 0.55 (95% CI: 0.36–0.86; *p* = 0.009)) predicted lower risk of a raised f-Hb and deprivation (SIMD 1-2: OR, 2.12 (95% CI: 1.38–3.25; *p* = 0.001)) predicted a higher risk of raised f-Hb. On multivariate analysis, bowel screening age (OR, 0.56 (95% CI: 0.36–0.89; *p* = 0.013)) and deprivation (SIMD 1-2: OR 2.13 (95% CI: 1.38–3.29; *p* = 0.001)) retained significance as independent predictors of lower and higher risk of a raised f-Hb, respectively.

**Table 4.** Univariate and multivariate binary logistic regression of factors associated with f-Hb ≥ 10 μg/g by significant bowel disease, other pathology and no pathology groups.

			Univariate			Multivariate		
			OR	95% CI	*p*	OR	95% CI	*p*
Significant bowel disease (*n* = 223)	Age (years)	< 50	1.00					
		50–74	0.42	0.15–1.17	0.097			
		≥ 75	1.16	0.32–4.29	0.821			
	Sex	Male	1.00					
		Female	0.94	0.46–1.93	0.865			
	Scottish Index of Multiple Deprivation	Non-deprived (SIMD 3–5)	1.00					
		Deprived (SIMD 1–2)	1.17	0.58–2.40	0.659			
	Oral anticoagulant	No	1.00					
		Yes	3.28	0.42–25.51	0.257			
	PPI	No	1.00					
		Yes	0.90	0.43–1.87	0.774			
	Rectal bleeding	No	1.00					
		Yes	3.63	1.66–7.97	0.001			
Other bowel disease (*n* = 682)	Age (years)	< 50	1.00					
		50–74	1.02	0.69–1.50	0.937			
		≥ 75	1.43	0.87–2.36	0.164			
	Sex	Male	1.00					
		Female	0.96	0.71–1.29	0.768			
	Scottish Index of Multiple Deprivation	Non-deprived (SIMD 3–5)	1.00					
		Deprived (SIMD 1–2)	1.32	0.98–1.78	0.072			
	Oral anticoagulant	No	1.00					
		Yes	1.60	0.82–3.12	0.169			
	PPI	No	1.00					
		Yes	1.60	1.15–2.11	0.004			
	Rectal bleeding	No	1.00					
		Yes	1.22	0.90–1.65	0.205			
No pathology (*n*=367)	Age (years)	< 50	1.00			1.00		
		50–74	0.55	0.36–0.86	0.009	0.56	0.36–0.89	0.013
		≥ 75	1.57	0.62–3.96	0.343	1.71	0.66–4.39	0.268
	Sex	Male	1.00					
		Female	0.88	0.57–1.37	0.578			
	Scottish Index of Multiple Deprivation	Non-deprived (SIMD 3–5)	1.00			1.00		
		Deprived (SIMD 1–2)	2.12	1.38–3.25	0.001	2.13	1.38–3.29	0.001
	Oral anticoagulant	No	1.00					
		Yes	2.28	0.71–7.33	0.166			
	PPI	No	1.00					
		Yes	1.25	0.82–1.91	0.293			
	Rectal bleeding	No	1.00					
		Yes	1.33	0.87–2.03	0.194			

## Discussion

To date, no studies have explored demographics independently associated with a raised f-Hb in symptomatic patients. In screener participants, a higher f-Hb independently correlates with older age, male sex, deprivation, smoking and use of aspirin, NSAIDs, oral anticoagulants, PPIs and antibiotics.^22–25^ In this study, we have shown higher f-Hb concentrations are seen in older symptomatic patients (≥ 75 years) but also in younger patients (< 50 years). This may be related to the impact of bowel cancer screening, with those aged 50–74 years with a raised f-Hb being more likely to be investigated via the screener pathway. On multivariate analysis, older age independently predicted a raised f-Hb (*p* = 0.050). While in the current study males did constitute a greater proportion of those with a raised f-Hb (males accounted for 43.9% of all participants, 48.1% of those with f-Hb ≥ 400 μg/g and 53.9% of those with f-Hb 150–399 μg/g), this did not reach statistical significance (*p* = 0.080). In agreement with studies investigating screener participants, this study has shown deprivation (*p* = 0.004) and oral anticoagulants (*p* = 0.017) to be associated with higher f-Hb, and these retained significance on multivariate analysis (*p* < 0.001 and *p* = 0.046). Patients on PPIs were more likely to have a raised f-Hb, but only between 10 and 399 μg/g (*p* = 0.007). No associations between NSAIDs or aspirin and f-Hb were detected.

Several studies have investigated the use of FIT for the diagnosis of significant bowel disease (cancer, advanced adenoma or IBD) in symptomatic patients. McDonald et al.^14^ reported on 280 patients referred from primary care with lower GI symptoms. They found that those with significant bowel disease had a median f-Hb of 15 μg/g which was significantly higher than those without (*p* < 0.0001). Additionally, patients with low-risk adenoma had a raised median f-Hb of 13 μg/g. In a similar study by Godber et al.,^15^ of 484 symptomatic patients, 45 had significant bowel disease; 196, low-risk adenoma, hyperplastic polyps, diverticular disease or haemorrhoids; and 243 patients had normal examinations. Median f-Hb for each group was 113 μg/g, 3 μg/g and 2 μg/g, respectively (*p* < 0.0001)

We have confirmed that in addition to colorectal cancer (*p* < 0.001), advanced adenoma (*p* < 0.001), non-advanced polyps (*p* < 0.001) and inflammatory bowel disease (*p* < 0.001) are all diagnoses independently associated with a raised f-Hb. We also found diverticulosis to correlate with a mildly raised f-Hb (10–149 μg/g, *p* < 0.001) and a notable association between a raised f-Hb and other lower GI malignancies (anal SCC or rectal lymphoma) (all 4 cases f-Hb ≥ 400, *p* < 0.001). Interestingly, while any advanced polyp (advanced adenoma or advanced sessile serrated polyp) predicted increased f-Hb on *χ*^2^ analysis (*p* < 0.001) and univariate binary logistic regression (*p* < 0.001), this did not retain significance on multivariate analysis. This most likely reflects the low number of advanced sessile serrated polyps in this study (*n* = 6) but may also relate to previous evidence suggesting that FIT is less sensitive for the detection of sessile serrated polyps as compared to adenoma, which may in part be explained by their frequent proximal colonic location.^36,37^

Several studies have previously examined factors correlating with FIT false positivity in screening participants. In the study by Ibanez-Sanz et al.,^25^ 89,199 bowel screening FITs from 46,783 patients were reviewed. False positivity was defined as f-Hb ≥ 20 μg/g without intermediate-risk, high-risk polyps or cancer. Independent predictors of false positivity were younger age (OR, 1.28 (95% CI: 1.12–1.46; *p* = 0.0002)), female sex (OR, 2.31 (95% CI: 2.03–2.64; *p* < 0.0001)), successive screening round (OR, 1.53 (95% CI: 1.35–1.74; *p* < 0.0001)), aspirin (OR, 1.30 (95% CI: 1.04–1.64; *p* = 0.02)), NSAID (OR, 1.48 (95% CI: 1.23–1.78; *p* < 0.0001)), PPI (OR 1.39 (95% CI: 1.18–1.65; *p* = 0.0001)), antibiotics (OR, 1.32 (95% CI: 1.03–1.71; *p* = 0.03)) and laxative (OR, 2.26 (95% CI: 1.06–4.80; *p* = 0.03)) use. Further studies have related false positivity in screening participants to both older age^29^ and younger age,^25,30^ female^25,26,30,32^ and male sex,^29^ smoking,^29^ high BMI,^29^ successive screening,^25,26^ the use of aspirin,^25^ NSAIDs,^25^ PPIs,^25,26,31^ antibiotics^25^ and laxatives,^25^ non-advanced adenomas,^27^ diverticular disease^27^ and anal pathology including haemorrhoids and anal fissures.^26,27,29^ De Klerk et al.^28^ performed a systematic review and meta-analysis of such studies and found younger age, female sex, NSAIDs, PPIs, anal fissures and peptic ulceration to be predictors of FIT false positivity in screener participants.

In the current study, we have established that deprivation is independently associated with a raised f-Hb in the absence of pathology at colonoscopy (*p* = 0.001). Mansouri et al.,^38^ a co-author of this study, found deprived individuals less likely to have cancer identified as a result of a positive FIT, within the Scottish Bowel Screening programme. It is interesting that this association with deprivation is shared by screening and symptomatic patients. In the review by Barnett et al.,^39^ they hypothesise that an elevated systemic inflammatory response (SIR) may explain the higher f-Hb concentrations observed in the absence of colorectal pathology, in screener participants with chronic conditions (ischaemic heart disease, cerebrovascular disease, diabetes and hypertension) and on certain medications (PPIs and anticoagulants). Perhaps a heightened SIR is one confounding variable which may link deprivation, co-morbidity and a raised f-Hb in the absence of colorectal pathology.

This study has a number of strengths. It is the first to perform multivariate analysis to establish independent predictors of a raised f-Hb in patients with lower GI symptoms. While this question has been applied to screener participants, it cannot be assumed that the same associations will be seen in symptomatic patients and indeed we have established several similarities and differences. Our study reflects real-life practice in the GG&C. Patients with both high- and low-risk symptoms and with and without rectal bleeding were included, reflecting the most up-to-date evidence^1,40–42^ and clinical use of FIT. Our study does however have limitations. It is retrospective in nature, and with the current sample size, it was difficult to establish clear associations between FIT and rarer diagnoses such as angiodysplasia, radiation proctitis, anal SCC, rectal prolapse and sessile serrated adenomas.

## Conclusion

We have found demographics including older age, deprivation and the use of oral anticoagulants to be independently associated with a raised f-Hb in patients with lower GI symptoms. In addition to colorectal cancer, advanced adenoma, non-advanced polyps, IBD, diverticulosis and anal SCC/rectal lymphoma are associated with a raised f-Hb. Deprivation is independently associated with a raised f-Hb in the absence of pathology. This should be considered when utilising FIT as part of a symptomatic referral pathway. Further work is required to establish why deprived patients are more likely to exhibit a raised f-Hb without pathology.
